# Potential utility of postmortem CT-based classification as a practical pre-autopsy assessment tool for hepatic steatosis

**DOI:** 10.1038/s41598-026-55019-5

**Published:** 2026-05-26

**Authors:** Ji-Hwan Park, Ji Su Han, Hyeong-Geon Kim, Jong-In Na

**Affiliations:** 1 Department of Forensic Medicine, National Forensic Service Seoul Institute, Seoul, 08063 Republic of Korea; 2 Department of Forensic Medicine, National Forensic Service Daejeon Institute, Daejeon, 34054 Republic of Korea

**Keywords:** Hepatic steatosis, Postmortem computed tomography, Autopsy, Histology, Forensic medicine, Diseases, Gastroenterology, Medical research

## Abstract

This study aimed to establish postmortem computed tomography (PMCT)-specific Hounsfield unit (HU) criteria and developed an HU-based assessment tool for pre-autopsy triage of hepatic steatosis. Overall, 166 deceased individuals underwent whole-body PMCT followed by autopsy with liver histopathology. Hepatic attenuation was measured as a volumetric mean HU of the entire liver and as a four-point mean HU. No significant difference was noted between the volumetric and four-point mean HU (*p* = 0.183). However, the four-point mean HU differed significantly across histological steatosis grades (*p* < 0.001) and decreased with increasing severity, although the difference between mild and moderate steatosis was non-significant (*p* = 0.981). Receiver operating characteristic analysis indicated an optimal cutoff of 42.59 HU (sensitivity 60.7%, specificity 86.1%), from which three HU-based categories were defined (≥ 44.5 HU: normal; 42.0–44.4 HU: borderline; <42.0 HU: suspected steatosis). In the validation cohort (*n* = 104), the tool achieved an accuracy of 0.865, sensitivity of 0.756, specificity of 0.949, precision of 0.919, and F1-score of 0.829. This three-category PMCT HU-based classification system provides a practical tool for the triage of hepatic steatosis. The high specificity and precision support its use as a rule-in aid to prioritize suspected steatosis cases and improve autopsy workflow efficiency.

## Introduction

Hepatic steatosis, also referred to as fatty liver disease (FLD), is an increasingly important global public health problem characterized by the excessive accumulation of triglycerides and other lipids within hepatocytes^1^. Hepatic fat is closely associated with metabolic syndrome and cardiovascular disease, and patients with non-alcoholic FLD (NAFLD) are at increased risk of coronary artery disease and atherosclerosis^2,3^. Recently, NAFLD has been redefined as metabolic dysfunction-associated steatotic liver disease^4,5^ to better reflect the systemic nature of the condition and its strong association with metabolic risk factors and cardiovascular morbidity^6^. Evaluation of hepatic steatosis is therefore meaningful not only from a clinical standpoint but also in the forensic context, where it may help explore potential contributions to the cause of death. The quantitative evaluation of hepatic steatosis has traditionally relied on liver biopsy; however, its invasiveness and risk of potentially fatal complications limit its repeated use^7^. Accordingly, there is a growing interest in the use of noninvasive imaging techniques to assess hepatic steatosis, and liver attenuation measured in Hounsfield units (HU) on computed tomography (CT) has been reported to show a quantitative correlation with hepatic fat content, making it a widely available and highly reproducible method^8,9^. However, direct comparison between clinical CT and postmortem CT (PMCT) is difficult owing to differences in imaging conditions and postmortem changes such as putrefaction and biochemical degradation, which can alter the radiodensity of hepatic tissue^10,11^. PMCT is a valuable tool in forensic practice worldwide, particularly in pre-autopsy screening^12^. While HU thresholds for diagnosing fatty liver are well-established in clinical medicine, the same thresholds cannot be directly adopted for PMCT owing to the aforementioned postmortem changes. Furthermore, although some PMCT-based studies have been conducted in other populations^13,14^, applying these external criteria to Korean individuals may yield inaccurate results, considering ethnic differences in body composition and metabolic profiles. However, in Korea, standardized HU-based criteria for quantitatively grading the severity of hepatic steatosis on postmortem CT images have not yet been established.

Therefore, the primary objective of this study was to develop and independently validate a practical PMCT-based assessment tool for pre-autopsy triage of hepatic steatosis, focusing on distinguishing the presence of any steatosis from normal liver. To achieve this, the study addressed three specific aims: (1) to compare the agreement and feasibility of volumetric versus four-point HU measurement methods; (2) to explore the association between four-point HU values and histological steatosis grades to derive an optimal HU cutoff; and (3) to validate the diagnostic performance of a simplified three-category HU-based tool in an independent cohort.

## Methods

### Study design and population

This retrospective study analyzed cases of consecutive forensic autopsy performed by a single forensic pathologist at the Seoul Institute of the National Forensic Service. The derivation cohort consisted of cases examined between 2022 and 2023, while the independent validation cohort included cases examined from 2024 to the first half of 2025.

**Fig. 1:**
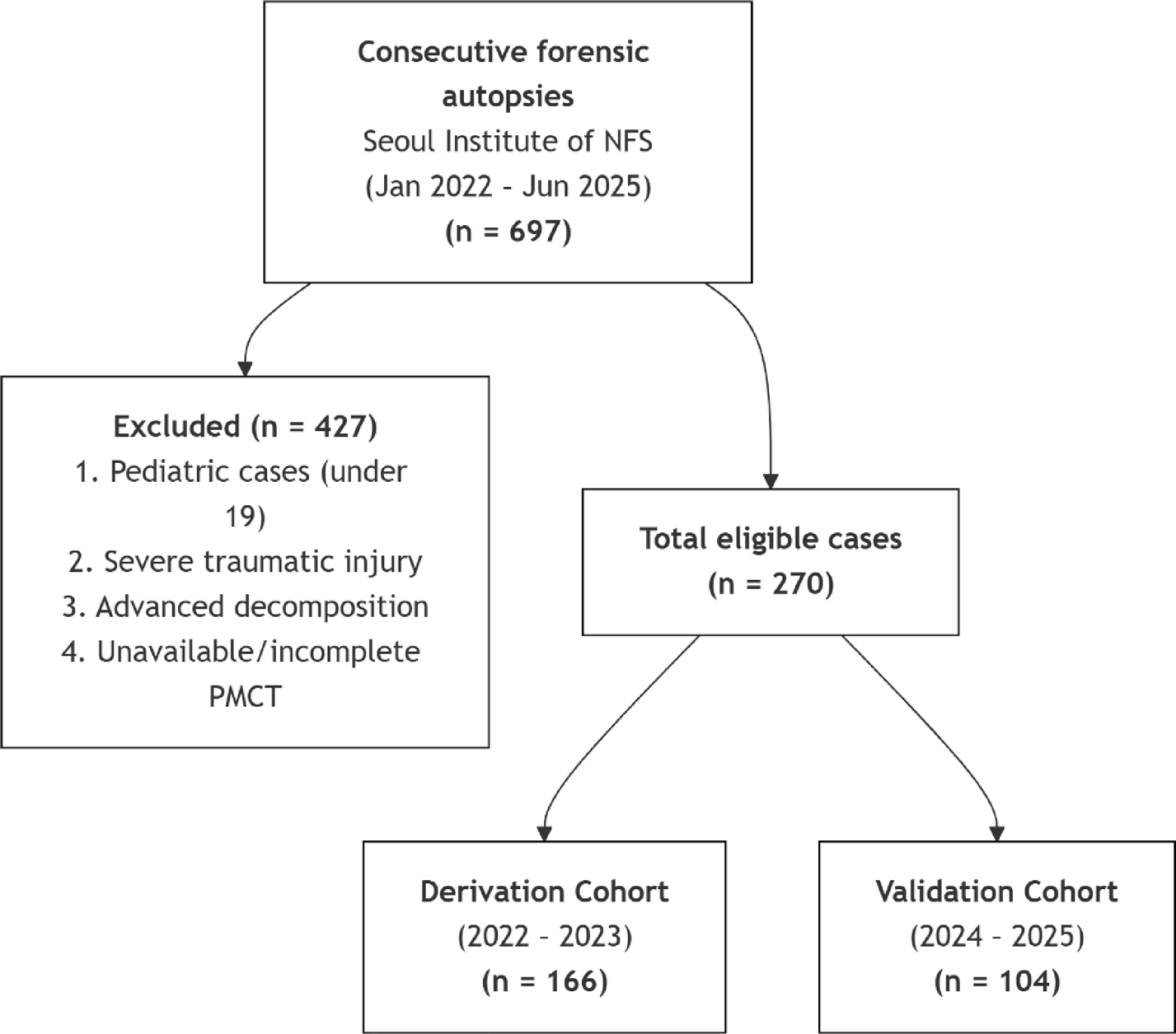
Flow diagram of the study. After applying the exclusion criteria, the derivation and independent validation cohorts included 166 and 104 cases, respectively.

To ensure accurate hepatic attenuation measurement and histological correlation, the following exclusion criteria were applied: (1) age < 19 years (pediatric cases); (2) unnatural deaths (e.g., trauma, poisoning) or severe traumatic injury to the torso or liver affecting HU measurement; (3) any macroscopic signs of decomposition (to avoid gas artifacts); and (4) unavailability of PMCT images. Consequently, a total of 166 cases (111 men and 55 women) were included in the derivation cohort, and 104 cases (72 men and 32 women) were included in the validation cohort (Fig. 1). The baseline characteristics of both cohorts are summarized in Table 1. There was no significant difference in age or sex distribution between the derivation and validation cohorts (all *p* > 0.05).

**Table 1:** Baseline characteristics of the derivation and validation cohorts.

Characteristic	Derivation Cohort (*n* = 166)	Validation Cohort (*n* = 104)	*p*-value
Age (years), mean ± SD	54.4 ± 16.4	53.8 ± 17.7	0.781*
Sex, n (%)			0.689**
Male	111 (66.9%)	72 (69.2%)	
Female	55 (33.1%)	32 (30.8%)	
Histological steatosis grade, n (%)			
Normal (< 10%)	77 (46.4%)	59 (56.7%)	
Mild (10–50%)	45 (27.1%)	18 (17.3%)	
Moderate (50–75%)	15 (9.0%)	9 (8.7%)	
Severe (≥ 75%)	29 (17.5%)	18 (17.3%)	

*Calculated using the independent samples t-test.

**Calculated using the Pearson’s chi-square test.

This study was approved by the Institutional Review Board of the National Forensic Service (approval no. 906-250319-BR-002-01). All methods were performed in accordance with the relevant guidelines and regulations, such as the Declaration of Helsinki^15^. The requirement for informed consent was waived because all autopsy procedures at the National Forensic Service are performed under a court-approved warrant, and the study was retrospective in nature.

### Histopathological analysis

Histopathological evaluation of the liver was performed by a single experienced forensic pathologist who was blinded to the PMCT findings. The severity of hepatic steatosis was graded based on the percentage of hepatocytes containing fat vacuoles as follows: normal (< 10%), mild (10% to < 50%), moderate (50% to < 75%), and severe (≥ 75%). This categorization follows the standard operational protocol routinely utilized for postmortem evaluation at our forensic institution, which is a practical modification of the traditional clinical scoring system^16^.

### PMCT image analysis and HU measurement

Prior to the PMCT examination, all bodies were stored in refrigerated chambers at approximately 4 °C to minimize postmortem changes. Although the precise postmortem interval varied, PMCT scans were generally performed within 24 to 72 h of death or discovery, in accordance with the standard autopsy protocol of the institute. All PMCT examinations were performed without contrast enhancement using a SOMATOM AS+ scanner (Siemens Healthcare, Erlangen, Germany). The main acquisition parameters were 120 kVp, 210 mAs, reconstruction slice thickness 0.75 mm, pitch 0.35, reconstruction increment 0.7 mm, and gantry rotation time 0.3 s. Whole-body scans were obtained, and the scan time ranged from 60 to 75 s depending on body length. Hepatic HU values were measured using two methods: (1) the volumetric mean HU of the whole liver and (2) four-point mean HU measurement.

### Volumetric mean HU of the entire liver

**Fig. 2:**
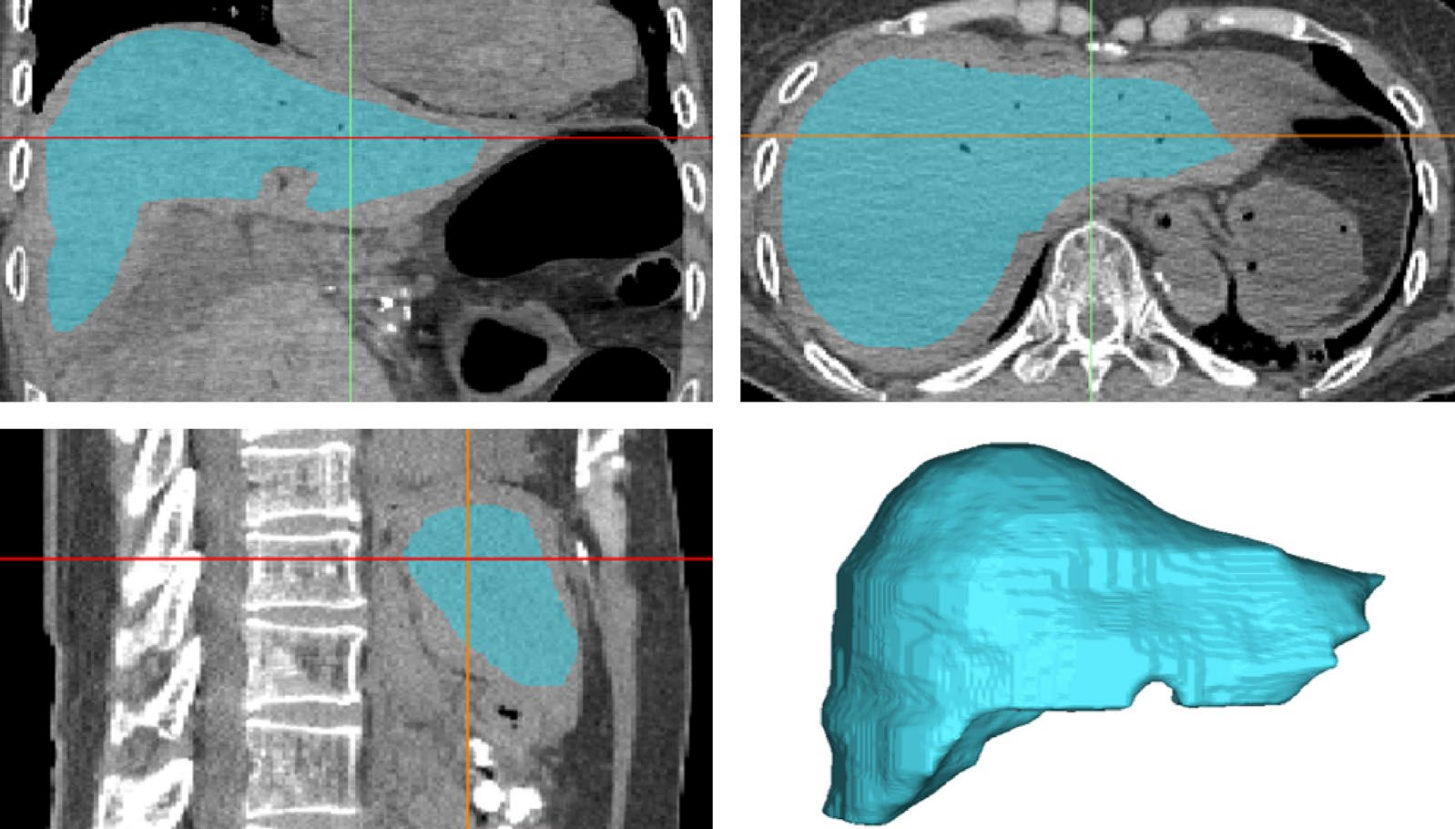
Three-dimensional reconstruction of the whole liver on postmortem computed tomography using Mimics.

Using Mimics 26.0 (Materialise Interactive Medical Image Control System, Materialise NV, Leuven, Belgium), the entire hepatic parenchyma was three-dimensionally segmented on the PMCT images, and the volumetric mean HU of the whole liver was calculated (Fig. 2).

### Four-point mean HU measurement

**Fig. 3:**
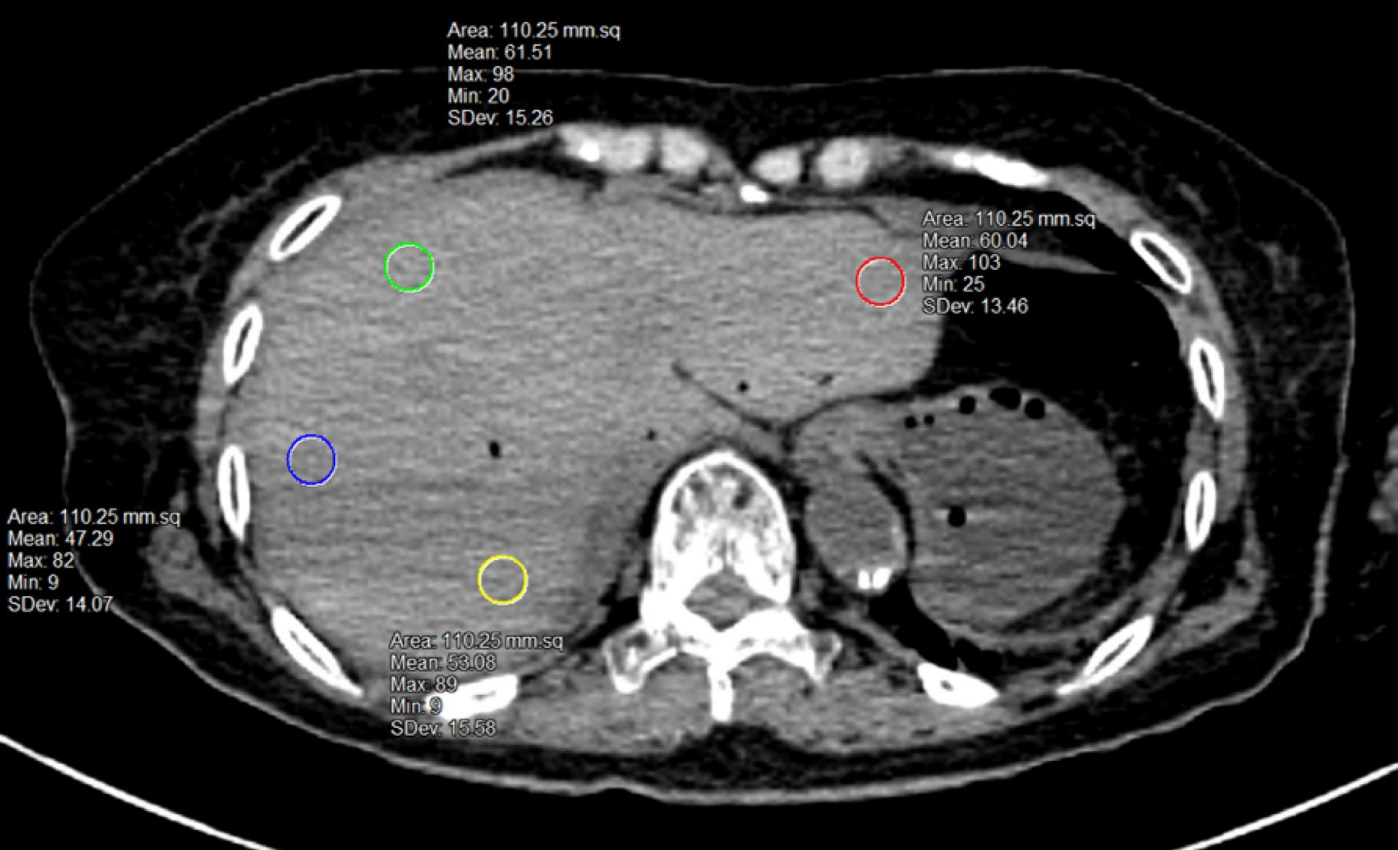
Four-point region-of-interest placement on postmortem computed tomography images using a thin-client viewer.

Using axial images on the AquariusNET Thin Client viewer (TeraRecon Inc., San Mateo, CA, USA), the axial slice demonstrating the largest cross-sectional area of the liver was selected to ensure sufficient parenchymal sampling. On this slice, four circular regions of interest (ROIs) were placed within the hepatic parenchyma while avoiding major vessels, bile ducts, the liver margin, and obvious artifacts (three ROIs in the right lobe and one in the left lobe). The ROI area was maintained between 100 and 120 mm², and the four measurements were averaged to obtain the four-point mean HU (Fig. 3). The measurements were performed by two radiologic technologists with over 5 and 10 years of experience, respectively. Inter-observer variability was not assessed in this study.

### Development of an auxiliary PMCT-based assessment tool for hepatic steatosis

#### Statistical analysis

Statistical analyses were performed using Jamovi (version 2.6.44) and Python in Google Colab (packages: NumPy 2.0.2, pandas 2.2.2, SciPy 1.16.1, scikit-learn 1.6.1, and Matplotlib 3.10.0). Baseline characteristics between the derivation and validation cohorts were compared using the independent samples t-test for continuous variables and Pearson’s chi-square test for categorical variables. The Shapiro–Wilk test was used to assess the normality of the HU values obtained from the two measurement methods (Mimics-based volumetric mean and four-point mean). Differences between the two measurement methods were assessed using the paired t-test for normally distributed variables and the Wilcoxon signed-rank test for non-normally distributed variables.

Agreement between the two measurement methods was evaluated using Bland–Altman analysis, from which the mean bias and 95% limits of agreement (LoA) were derived. In addition, the concordance correlation coefficient (CCC) with its 95% confidence interval was calculated to quantify the level of concordance.

#### Comparison with histopathological findings and receiver operating characteristic analysis

HU values were compared across histopathological grades of hepatic steatosis (normal, mild, moderate, and severe). Reflecting the results of the normality tests, the Kruskal–Wallis test was used for comparisons, followed by pairwise post hoc comparisons. The distribution of HU values according to the degree of steatosis was visualized using boxplots. Histopathological findings were then dichotomized into “normal” and “steatosis” (including all grades of steatosis). To determine the optimal four-point mean HU cutoff value for discriminating the presence of steatosis, receiver operating characteristic (ROC) analysis was performed in Python. The area under the curve (AUC), sensitivity, and specificity were calculated. The optimal cutoff value was determined using the Youden index (maximum value of sensitivity + specificity − 1). For ease of interpretation and potential practical use, we then constructed a three-tier classification scheme (“normal,” “borderline,” and “suspected steatosis”) based on this optimal cutoff.

### Validation of the auxiliary assessment tool

**Fig. 4:**
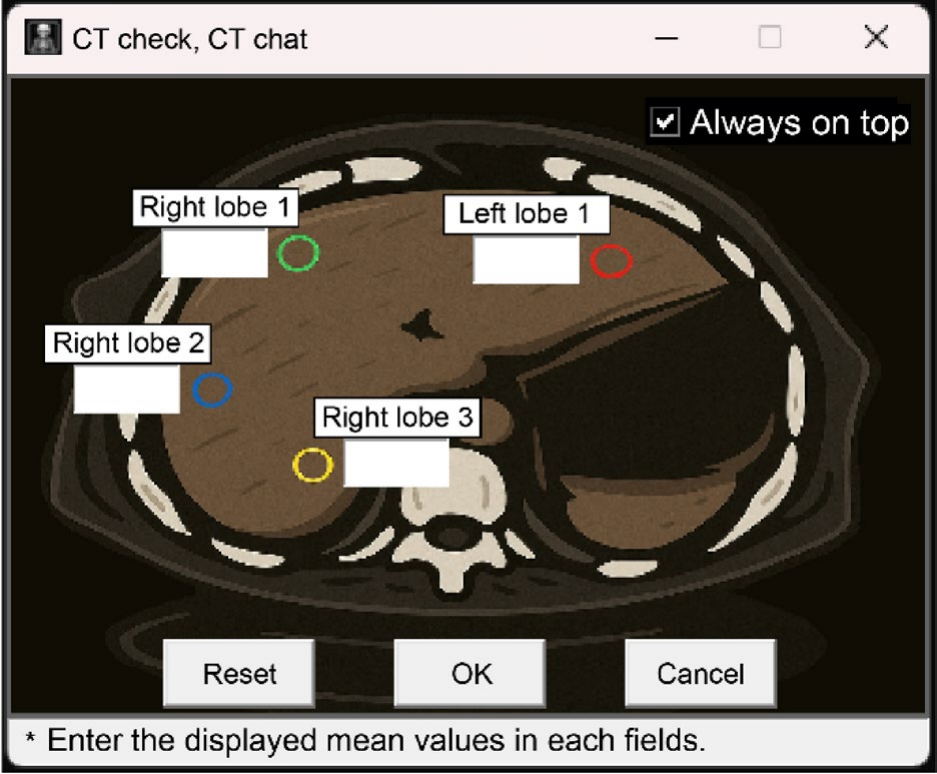
Screenshot of the Python-based auxiliary assessment tool for hepatic steatosis.

Based on the ROC analysis, we developed a Python-based auxiliary assessment tool that outputs three categories—“normal,” “borderline”(needs further evaluation), and “steatosis”—when a hepatic HU value is entered (Fig. 4). To evaluate the utility of this tool, the four-point mean HU values of 104 cases in the independent validation cohort from 2024–2025 were entered into the program. The tool’s classifications were then compared with the actual histopathological liver findings. Because the primary aim of this study was to distinguish the presence of steatosis, cases classified as “borderline” were regarded as “non-steatosis” when constructing a 2 × 2 confusion matrix. On this basis, we calculated the accuracy, sensitivity, specificity, precision, and F1-score of the tool.

## Results

### Comparison and agreement between the two HU measurement methods

**Fig. 5:**
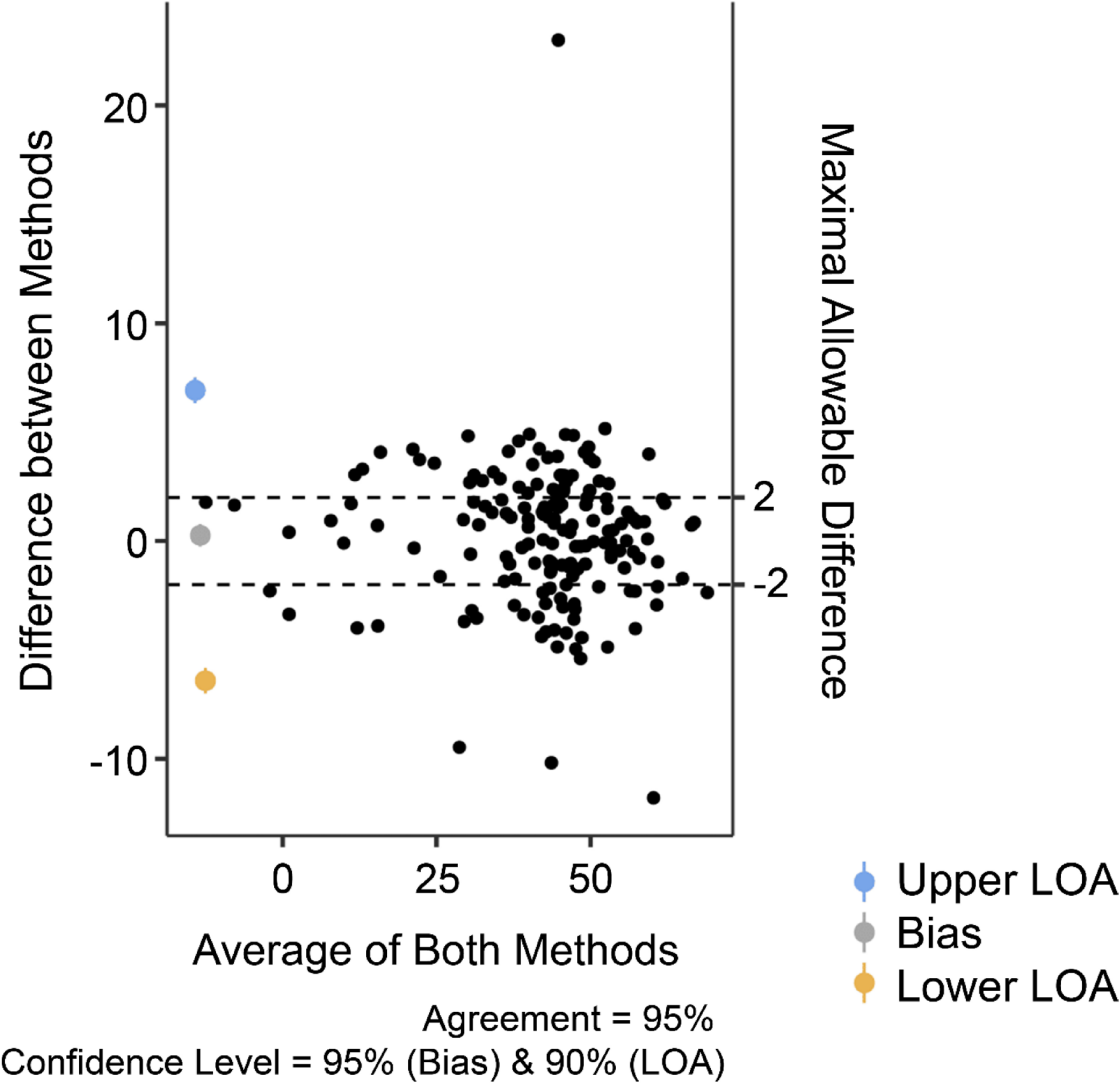
Bland–Altman plot showing the agreement between the two hepatic HU measurement methods. Abbreviations: Hounsfield unit (HU), limit of agreement (LoA).

The mean HU values obtained from the Mimics-based volumetric measurement and the four-point measurement were non-normally distributed (*p* < 0.05). There was no significant difference between the Mimics-based mean HU and the four-point mean HU (*p* = 0.183). The effect size (r) was 0.119, indicating a small effect and suggesting that the two methods showed overall agreement. In the Bland–Altman analysis, the mean difference (mean bias) between the two methods was minimal at 0.256 HU, and the 95% LoA ranged from − 6.410 HU to + 6.922 HU (Fig. 5). The CCC was 0.972, indicating a very high level of concordance between the two measurement methods.

### Association between histopathological findings and four-point mean HU values

Given the absence of a significant difference and the high level of agreement between the two HU measurement methods, subsequent analyses were performed using the four-point mean HU, which is more convenient for practical application. There was a significant difference in the four-point mean HU values across histopathological grades of hepatic steatosis (normal, mild, moderate, and severe; χ^2^(3) = 63.0, *p* < 0.001). Post hoc pairwise comparisons showed that the normal group differed significantly from the mild (*p* = 0.010), moderate (*p* = 0.007), and severe (*p* < 0.001) steatosis groups. The severe steatosis group also differed significantly from all other groups (normal, mild, and moderate; all *p* < 0.001). In contrast, no significant difference was observed between the mild and moderate steatosis groups (*p* = 0.981). These results are summarized in Table 2.

**Table 2:** Pairwise comparisons of liver HU (four-point method) among histological steatosis grades.

Comparison	W	*p*
Mild steatosis vs. Moderate steatosis	-0.543	0.981
Mild steatosis vs. Normal	4.401	0.010
Mild steatosis vs. Severe steatosis	-7.743	< 0.001
Moderate steatosis vs. Normal	4.537	0.007
Moderate steatosis vs. Severe steatosis	-6.005	< 0.001
Normal vs. Severe steatosis	-10.133	< 0.001

Abbreviations: Hounsfield unit (HU).

**Fig. 6:**
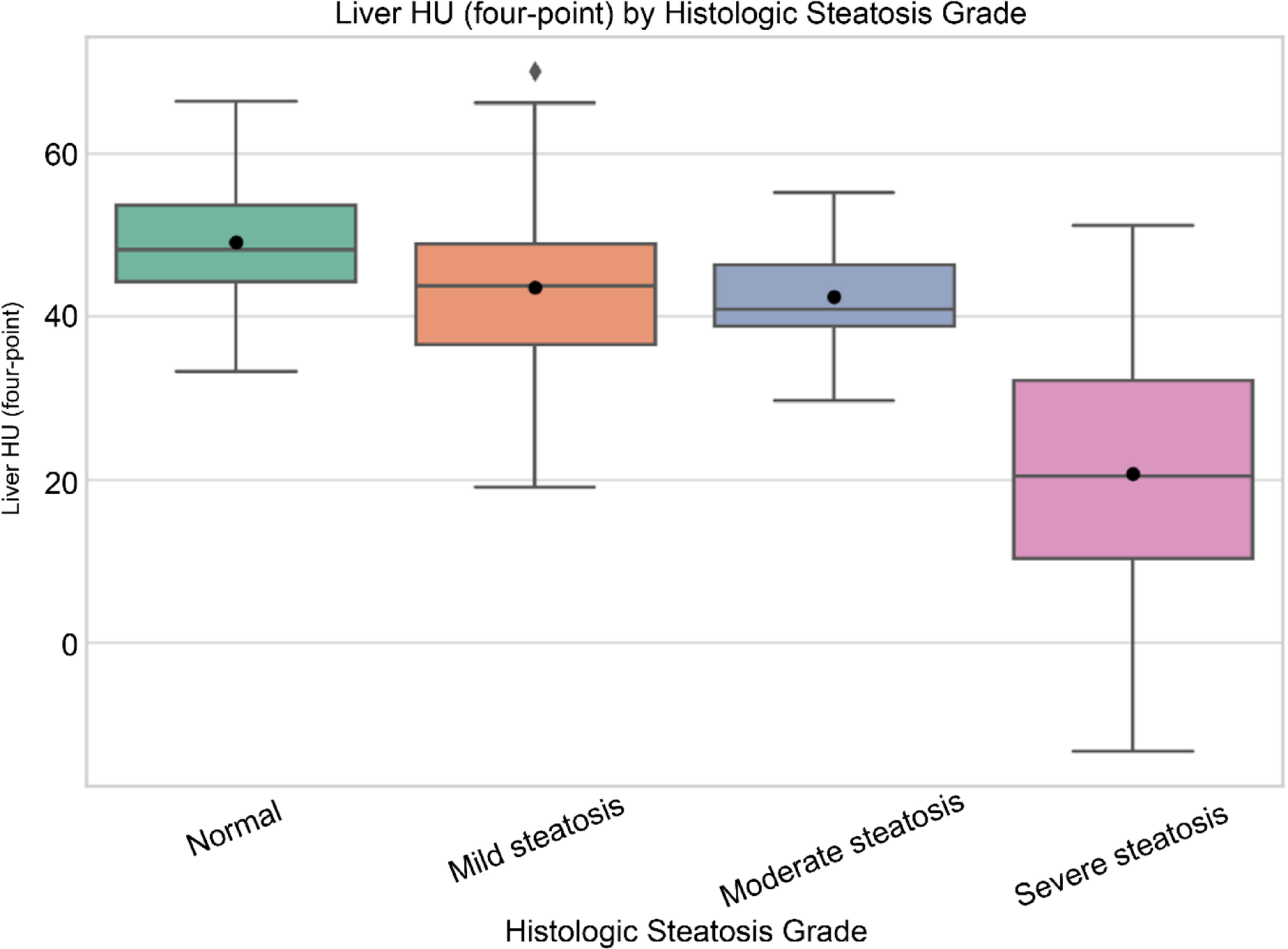
Liver attenuation (four-point HU) by histologic steatosis grade. Abbreviations: Hounsfield unit (HU).

As the severity of hepatic steatosis increased, the four-point mean HU showed a significant decreasing trend, as illustrated in Fig. 6.

### ROC analysis and establishment of a diagnostic cutoff for hepatic steatosis

**Fig. 7:**
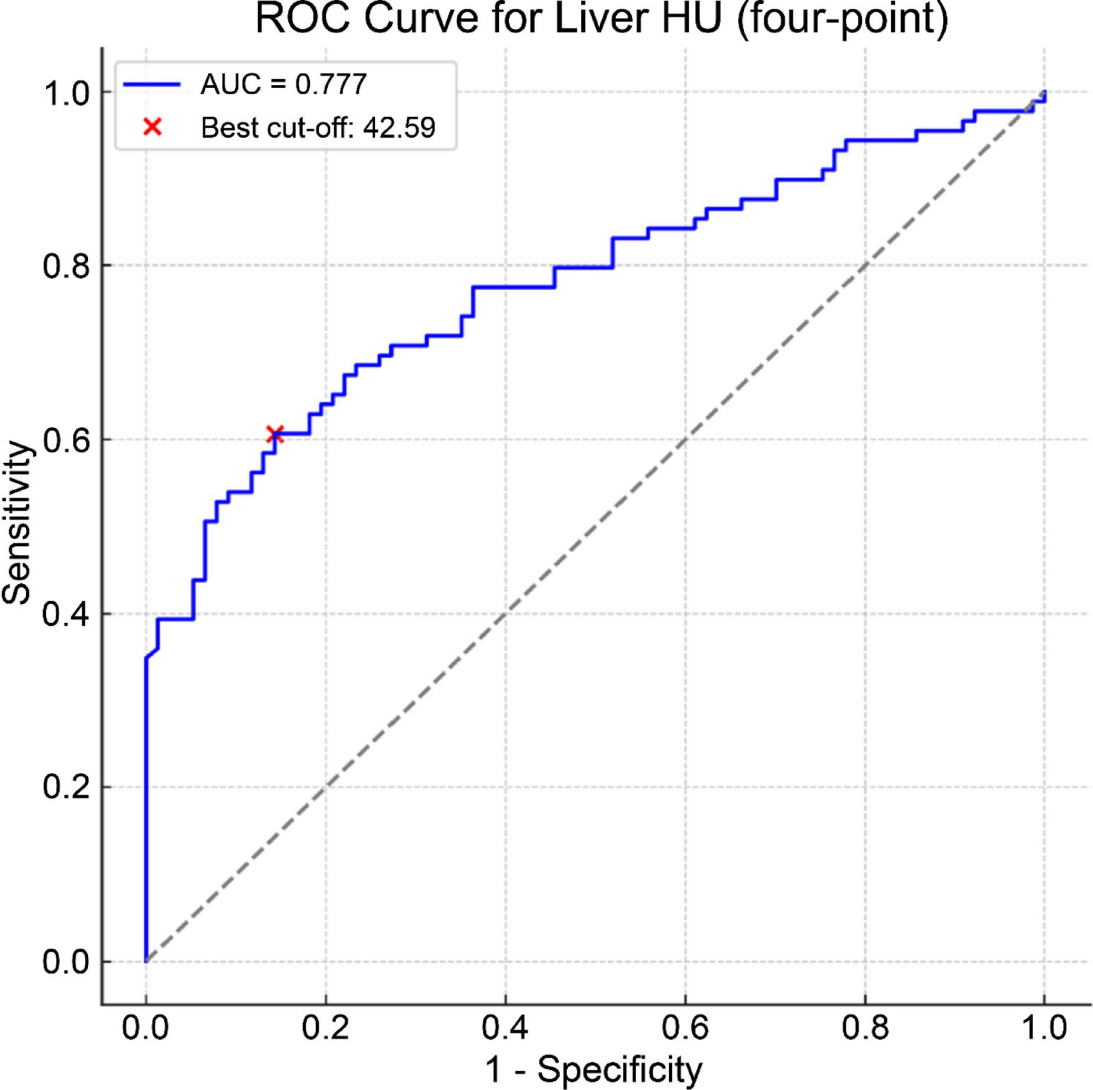
Receiver operating characteristic curve of liver attenuation (four-point HU) for distinguishing histologic normal liver from fatty liver (≥ mild steatosis). Abbreviations: Hounsfield unit (HU).

To distinguish histologically normal livers from those with steatosis (any grade), ROC analysis was performed using the four-point mean HU as the test variable. The ROC analysis yielded an AUC of 0.777. The optimal cutoff value for differentiating a normal liver from steatosis was 42.59 HU, which corresponded to a sensitivity of 60.7% and a specificity of 86.1% (Fig. 7).

Based on the ROC-derived optimal cut-off of 42.59 HU, we defined the following three HU-based categories for liver CT:

- HU of ≥ 44.5: Normal.
- HU of 42.0–44.4 HU: Borderline.
- HU of < 42.0 HU: Suspected steatosis.

### Validation of the auxiliary assessment tool for hepatic steatosis

The performance of the auxiliary assessment tool, developed using the above three-category HU classification, was evaluated in an independent validation cohort of 104 cases. In this validation analysis, cases in the “borderline” category were classified as non-steatosis. Based on this binary classification, the confusion matrix was as follows: true positive = 34, false positive = 3, false negative = 11, and true negative = 56 (Table 3).

**Table 3:** Confusion matrix of the HU-based three-category auxiliary assessment tool for detecting histological fatty liver in the independent validation cohort (*n* = 104), with “borderline” cases classified as non-fatty liver.

Predicted vs. reference	Steatosis (+)	Normal (−)	Total
Predicted steatosis (+)	34 (TP)	3 (FP)	37
Predicted non-steatosis (−)	11 (FN)	56 (TN)	67
Total	45	59	104

Abbreviations: Hounsfield unit (HU), true positive (TP), false positive (FP), true negative (TN), false negative (FN).

Based on these values, the performance metrics of the tool were as follows: accuracy, 0.865; sensitivity, 0.756; specificity, 0.949; precision, 0.919; negative predictive value, 0.836; and F1-score, 0.829. Detailed 95% confidence intervals for these metrics are provided in Table 4.

**Table 4:** Diagnostic performance of the HU-based three-category auxiliary assessment tool for hepatic steatosis in the independent validation cohort (*n* = 104), with 95% confidence intervals.

Metric	Value	95% CI
Accuracy	0.865	0.787–0.918
Sensitivity	0.756	0.613–0.858
Specificity	0.949	0.861–0.983
Precision	0.919	0.787–0.972
Negative Predictive Value	0.836	0.744–0.928
F1-score	0.829	—

Abbreviations: Hounsfield unit (HU), confidence interval (CI).

## Discussion

To the best of our knowledge, this is the first study to propose PMCT-based hepatic steatosis criteria specifically tailored to the Korean population and to rigorously verify their diagnostic performance using an independent temporal validation cohort. Our findings revealed that the relatively simple four-point HU measurement method did not significantly differ from the more sophisticated 3D segmentation–based measurement and demonstrated a high level of concordance (CCC = 0.972), confirming its practical utility. Further, we successfully established a three-tier HU-based classification tool (“normal,” “borderline,” and “suspected steatosis”) which, when evaluated in an independent validation cohort, achieved an accuracy of 0.865 and an F1-score of 0.829. The tool also had particularly high specificity (0.949) and precision (0.919), supporting its potential usefulness as a triage tool in forensic practice.

The first major contribution of this study is establishing the reliability of a simple four-point ROI measurement method for PMCT-based liver attenuation analysis. Although whole-liver 3D segmentation and calculation of volumetric mean HU using dedicated software such as Mimics may offer high accuracy, it is time-consuming and requires access to specialized programs. In contrast, the four-point method can be performed rapidly on standard PACS viewers. The minimal mean difference of 0.256 HU between the two methods (*p* = 0.183) and the very high concordance (CCC = 0.972) observed in this study suggest that, in the time-pressured environment of forensic practice, the four-point measurement method can adequately substitute for full 3D volumetric measurement. While the 95% limits of agreement (LoA) between the two methods spanned approximately ± 6.5 HU—which is wider than our defined borderline category (42.0–44.4 HU)—it is important to note that our diagnostic thresholds were derived directly from the four-point measurements against the true histopathological reference standard, rather than being extrapolated from the 3D volumetric data. Therefore, the four-point method functions as an independent, standalone index, and its intrinsic diagnostic stability is maintained despite the LoA margin with the 3D method. In this study, it was crucial to establish PMCT-specific threshold values because attenuation criteria derived from clinical CT cannot be directly applied to postmortem images. Clinical studies have reported hepatic HU values of 60–70 HU in healthy children and adults^17^, which is markedly higher than the threshold of ≥ 44.5 HU used to define a “normal” liver in our PMCT cohort. This discrepancy can be interpreted as reflecting factors such as premortem nutritional status and fasting duration, as well as postmortem glycogenolysis, which leads to the depletion of hepatic glycogen and consequently lower overall liver attenuation on PMCT compared with clinical CT. Therefore, normal reference values from clinical CT cannot simply be transferred to PMCT, and PMCT-specific threshold values, such as those proposed in this study, are essential.

Against this background, this study analyzed the association between histopathological findings and four-point mean HU values to address the lack of PMCT-based criteria for grading hepatic steatosis in Korea. Our analysis demonstrated a significant difference in HU values across steatosis grades (*p* < 0.001), but post hoc comparisons revealed no significant difference between mild and moderate steatosis (*p* = 0.981). This finding indicates that HU values alone have limited ability to clearly distinguish between finer grades of steatosis, which is consistent with observations from clinical CT studies^18^. Therefore, we focused on discriminating the presence or absence of steatosis rather than on detailed subclassification. Based on the optimal cutoff value derived from ROC analysis (42.59 HU, AUC = 0.777), we proposed a three-category classification system (“normal” [≥ 44.5 HU], “borderline” [42.0–44.4 HU], and “suspected steatosis” [< 42.0 HU]), which represents a reasonable approach to reducing ambiguity and enhancing practical applicability. The introduction of a “borderline” category may function as a buffer to minimize false negatives and to prompt further evaluation in clinical or forensic settings.

The third and most important finding of this study is that the three-category auxiliary assessment tool demonstrated high utility in an independent validation cohort of 104 cases. In our evaluation, we adopted a strict approach by treating the “borderline” category as non-steatosis. Under this condition, the tool yielded very high specificity (0.949) and precision (0.919), indicating that its negative classifications (“normal” or “borderline”) are highly reliable and that cases categorized as “suspected steatosis” have a very high probability of truly having steatosis. In contrast, the sensitivity (0.756) was relatively lower, meaning that approximately 24.4% of cases with histologically confirmed steatosis were classified as “normal” or “borderline.”

However, considering the intended role of the tool as a pre-autopsy triage aid, its value as a rule-in instrument is substantial; by prioritizing cases flagged as “suspected steatosis” for more intensive histopathological examination, it can help improve the efficiency of autopsy workflows. The F1-score of 0.829 further indicates a favorable balance between sensitivity and precision. In real-world forensic settings, autopsies are often performed under considerable time constraints^19^, and the rapid pre-autopsy triage of PMCT images for suspected hepatic steatosis can provide practical support in determining which additional pathological examinations are necessary.

Another important consideration is the epidemiological and anatomical characteristics of hepatic steatosis. Consistent with previous literature demonstrating that metabolic dysfunction-associated steatotic liver disease (MASLD) affects men more frequently than women^20^, our study population showed a male predominance. However, because our PMCT classification relies on objective physical tissue attenuation (HU), this sex difference in prevalence does not affect the diagnostic accuracy of the thresholds. Furthermore, while conventional autopsy practice relies on localized liver biopsy, which may be subject to sampling errors due to the heterogeneous distribution of steatosis within the liver^21^, our four-point ROI CT method can partially mitigate this spatial heterogeneity by sampling and averaging multiple regions of the hepatic parenchyma.

This study has several strengths. First, it is one of the pioneering efforts to propose PMCT-based HU criteria for hepatic steatosis tailored to the Korean forensic setting. Second, hepatic HU values were analyzed against a clear reference standard, namely the histopathological examination of liver tissue. Third, by separating the derivation cohort (*n* = 166) from an independent validation cohort (*n* = 104), we were able to objectively assess the generalizability of the proposed tool, thereby enhancing the robustness of our findings.

Despite these strengths, this study has some limitations. First, this was a single-center retrospective study based on data from the Seoul Institute of the National Forensic Service, and the proposed criteria may not be directly applicable to other institutions that use different CT scanners or imaging protocols. Second, as noted in the Introduction, PMCT findings can be influenced by postmortem changes such as decomposition and biochemical degradation^10,11^. Although we refrigerated the bodies and excluded visibly decomposed cases, the postmortem interval (PMI) of our cohorts ranged up to 72 h, which is wide enough for substantial biological variability to occur. Because we lacked standardized radiological decomposition scores (e.g., RA-Index) or strict temperature controls, our proposed HU cutoff values may partially reflect this uncontrolled postmortem variability rather than pure steatosis. Third, our evaluation strategy, in which the “borderline” category was treated as non-steatosis, was a deliberate, conservative choice that directly contributed to the high specificity (0.949) and precision (0.919) of the tool. By minimizing false-positive results, this approach maximizes the tool’s ‘rule-in’ utility, ensuring that cases flagged as ‘suspected steatosis’ are prioritized with high confidence in the forensic triage workflow. While this inevitably leads to a trade-off with lower sensitivity, it is highly appropriate for our intended triage and prioritization purposes. Detailed pathological characterization of the borderline group should be addressed in future studies. Fourth, due to the retrospective nature of this forensic study, we were unable to obtain reliable data on Body Mass Index (BMI) for all cases. In the postmortem setting, accurate measurement of height and weight can be challenging, and such information is often missing from police or medical examiner reports. Although BMI is a well-known risk factor for hepatic steatosis in clinical settings, our study focused on the direct physical measurement of liver attenuation (HU), which provides an objective assessment of the organ’s state regardless of the individual’s external body habitus. Finally, although HU measurements were performed by experienced radiologic technologists, inter-observer variability was not formally assessed, which remains a limitation to be addressed in future studies. However, unlike subjective morphological evaluations, our PMCT evaluation utilized a standardized four-point ROI method that extracts objective physical attenuation (HU) values, inherently minimizing observer-dependent bias.

Given the simplicity of the four-point measurement method, the technique is straightforward to learn and implement, offering significant educational value for training forensic practitioners. From a practical standpoint, integrating this standardized triage and prioritization tool into routine pre-autopsy workflows can facilitate rapid decision-making and help prioritize suspected cases effectively. Future research should focus on multicenter validation to further establish the broader applicability of these criteria.

In conclusion, this study successfully validated a three-category classification scheme (normal, borderline, and suspected steatosis) based on four-point PMCT HU measurements as a useful auxiliary tool for the rapid triage of hepatic steatosis in forensic practice. In particular, the high specificity and precision suggest that this tool may be effective for prioritizing suspected cases, while the negative predictive value of 0.836 supports its utility in ruling out significant steatosis. Nevertheless, it should be noted that this tool is designed for triage and prioritization rather than as an independent diagnostic method for determining the cause of death. Furthermore, as the proposed criteria are derived from a specific Korean population, local validation is recommended before routine implementation in other forensic institutions. Future multicenter, large-scale prospective studies are warranted to further verify the generalizability of these findings. In addition, it would be valuable to confirm whether the clinical observation that normal hepatic attenuation is consistently higher than splenic attenuation (typically by 8–10 HU)^22^ also holds true in the PMCT setting and to explore combining this relative liver–spleen difference with absolute HU criteria to improve steatosis discrimination. Furthermore, developing predictive algorithms that incorporate other potential confounding variables affecting hepatic HU values—such as the postmortem interval and cause of death—could further enhance diagnostic accuracy in subsequent studies.

## Data Availability

The data that support the findings of this study are not publicly available due to legal and privacy restrictions of the National Forensic Service but may be available from the corresponding author on reasonable request and with permission of the National Forensic Service.

## Code availability

The Python code used for the auxiliary assessment tool is available from the corresponding author upon reasonable request.
